# Trends in human leptospirosis in Denmark, 2012-2021

**DOI:** 10.3389/fcimb.2023.1079946

**Published:** 2023-02-13

**Authors:** Caroline Eves, Charlotte Kjelsø, Guido Benedetti, Charlotte Sværke Jørgensen, Karen Angeliki Krogfelt

**Affiliations:** ^1^ Department of Infectious Disease Epidemiology and Prevention, Statens Serum Institut, Copenhagen, Denmark; ^2^ Department of Virus and Microbiological Special Diagnostics, Statens Serum Institut, Copenhagen, Denmark; ^3^ Department of Science and Environment, PandemiX Center, Roskilde University, Roskilde, Denmark

**Keywords:** leptospirosis, Denmark, disease surveillance, zoonosis, emerging disease, one health (OH)

## Abstract

Leptospirosis is a zoonotic bacterial infection that can cause influenza-like symptoms and severe disease. In Denmark, leptospirosis is rare, non-endemic, and most commonly transferred to humans from mice and rats. Cases of human leptospirosis in Denmark are by law notifiable to Statens Serum Institut. This study aimed to describe trends in incidence of leptospirosis in Denmark from 2012 to 2021. Descriptive analyses were used to calculate the incidence, geographical distribution and possible routes of infection, as well as testing capacity and serological trends. The overall incidence rate was 0.23 per 100,000 inhabitants, with the highest annual incidence of 24 cases in 2017. Men between 40-49 years old were the demographic group most commonly diagnosed with leptospirosis. August and September were the months with highest incidence over the entire study period. The most common serovar observed was Icterohaemorrhagiae, although over a third of cases were diagnosed *via* polymerase chain reaction alone. The most common sources of exposure reported were travel abroad, farming, and recreational contact with fresh water, the latter being a new exposure compared to previous studies. Overall, a One Health approach would ensure better detection of outbreaks and milder disease. Additionally, preventative measures should be expanded to include recreational water sports.

## 1 Introduction

Leptospirosis is a zoonotic infectious disease caused by *Leptospira*, a pathogenic bacteria (World Health Organization, 2003). There are an estimated 1.3 million new cases of human leptospirosis, hereafter referred to as leptospirosis, diagnosed globally each year (Costa et al., 2015). Leptospirosis predominately impacts populations living in areas with tropical climates, with outbreaks occurring most frequently after rain storms and episodes of flooding (Pan American Health Organization,).

There are many different species and strains of *Leptospira*. A recent study identified 64 different species of *Leptospira* (Vincent et al., 2019) and the World Health Organization (WHO) estimates that there are over 200 various serovars belonging to approximately 25 different serogroups (World Health Organization, 2003). These serovars are often unique to a particular species of host and can therefore be used to trace the likely source of infection in humans (van Alphen et al., 2015).

Infection with leptospirosis occurs *via* occupational or recreational exposure to animals or environmental elements, e.g. urine-contaminated fresh water and soil (Centers for Disease Control and Prevention and National Center for Emerging and Zoonotic Infectious Diseases (NCEZID), 2015). In Denmark, rats and mice are the primary hosts, though other animals, e.g. cattle, pigs and dogs, can transmit *Leptospira* to humans as well (Department of Infectious Disease and Prevention, 2020). Vaccination for leptospirosis in humans is not offered in Denmark, but is available for dogs and certain production animals, such as swine and cattle (Danish Veterinary and Food Administration, 2019; Department of Infectious Disease and Prevention, 2020).

Leptospirosis can present with a wide array of symptoms, from non-specific flu-like symptoms often resembling other febrile infections to severe disease (Haake and Levett, 2015; Rajapakse, 2022). Weil’s disease, classified by jaundice and liver failure, is a severe form of leptospirosis, which can lead to myocarditis and multiple organ failure (Pan American Health Organization,; Haake and Levett, 2015). Furthermore, the development of Pulmonary Hemorrhage Syndrome leading to respiratory failure has been linked to higher mortality among leptospirosis patients, with a fatality rate of over 50% (Haake and Levett, 2015; Rajapakse, 2022).

Leptospirosis is non-endemic in Denmark, with an average of 17 cases per year from 1980 – 2012 (van Alphen et al., 2015). Although rare, leptospirosis is notifiable to the Department of Infectious Disease Epidemiology and Prevention, Statens Serum Institut (SSI) under Danish law (The Danish Health Authority, 2000).

Following the work by van Alphen et al. (2015), this study updates the descriptive epidemiological analysis of leptospirosis in Denmark in order to further examine trends of disease and protect susceptible population groups. The aim of this study was to describe the incidence, geographical distribution and possible routes of infection of human leptospirosis in Denmark from 2012-2021, as well as test capacity and serological trends, in order to propose appropriate health care and prevention advice.

## 2 Materials and methods

### 2.1 Laboratory diagnostics

SSI is the sole diagnostic laboratory for leptospirosis in Denmark. Since 2012, in order to comply with the WHO standards and compare results with other European reference laboratories, serovars included in the microagglutination test (MAT) were purchased from Academic Medical Center (AMC) University of Amsterdam, Netherlands (previously the Royal Tropical Institute in Amsterdam (KIT)). The serovars in the MAT included Autumnalis, Bataviae, Bratislava, Cynopteri, Canicola, Castellon, Copenhageni, Grippotyphosa type Moskva, Hardjo, Hebdomadis, Hurstbridge, Icterohaemorrhagiae, Javanica, Pomona, Sejroe and Tarassovi. Sera dilutions (in titer) of 30, 100, 300, 1,000, 3,000, 10,000, and 30,000 were tested by MAT and observed by dark field microscopy. The serogroup was then determined from the highest titer for one or more serovars in the MAT (van Alphen et al., 2015). Cross-reactions between serovars are known, therefore we report the infecting serogroups (Levett, 2003).

The sera were also tested against the non-pathogenic strain Patoc. Although Patoc is saprophytic, it is, together with appropriate concurrent clinical signs, considered an indicator for leptospirosis infection in Denmark, as it has been known to cross-react with various other pathogenic serovars (Picardeau, 2013). Follow up serology testing is recommended in the event of a reaction to only the Patoc serovar.

Polymerase chain reaction (PCR) testing was also performed at SSI. An in house qPCR-test with an improved method of DNA extraction, where blood cultures were subjected to an improved benzyl alcohol and column-based DNA extraction protocol as previously described by Villumsen et al. (2010), was used for detection of leptospirosis by amplification of *lipl32* and 16S rRNA genes (Villumsen et al., 2012). DNA was extracted from 100 microliters of the specimen using the Blood & Tissue Kit (Qiagen, Germany) by adding benzyl alcohol to the solution, and subsequently separated from the aqueous phase containing the DNA by centrifugation. The PCR primers Lepto F (5’171 CCCGCGTCCGATTAG 3’) and Lepto R (5’258 TCCATTGTGGCCGRA/GACAC3’) were located between the positions 171 and 258 of the rrs (16S) gene with an expected product size of 87 bp product. The probe [5’205(FAM) CTCACCAAGGCGACGATCGGTAGC228 3’ (TAMRA)]- PCR conditions included 50 cycles, each cycle consisting of 95°C for 15 seconds and 60°C for one minute. In the event of a positive PCR result, submission of additional samples was requested for further serological testing.

### 2.2 Case definition

A case was defined as a resident of Denmark at the time of infection with exposure and clinical assessment suggestive of leptospirosis infection and either a titer ≥100 for at least one diagnostic serovar or other laboratory results indicative of leptospirosis infection (i.e. Patoc, Hurstbridge and other serovars, PCR, and/or culture).

### 2.3 Data collection

Laboratory data for all cases of leptospirosis diagnosed in Denmark from January 1, 2012 to December 31, 2021 were retrieved from the Danish Microbiology Database (MiBa) (Voldstedlund et al., 2014). These data included laboratory address information, results for PCR and serologic testing, and test date.

Clinical notification data were collected from the Department of Infectious Disease Epidemiology and Prevention at SSI. These data included patient demographic information, timeframe of disease onset, hospital admission, vital status, data pertaining to the patient’s profession and/or workplaces, travel abroad in relation to infection, and any other relevant information referring to the suspected source of infection (Statens Serum Institut, 2022). Notification data also included patient address, which was used to calculate incidence rates by province. For non-notified cases, laboratory address was used to determine province. Additional information was extracted from the MiBa when available.

Danish population data from 2012-2021 by province, age group, and sex were extracted from Statistics Denmark (Statistics Denmark, 2012-2021).

### 2.4 Data analysis

Annual incidence rates per 100,000 were calculated using year-specific population figures as of July 1^st^. The population as of January 1, 2017, which was considered the midterm population, was used to calculate incidence rates by sex, age group, and province over the entire study period. Regarding source of infection, exposures were categorized into six groups: Work-related, Recreation, Travel Abroad, Sewage-related, Other and Unknown. Source of exposure by serovar was also investigated.

All data were analyzed within the Department of Infectious Disease Epidemiology and Prevention, SSI. Data cleaning and analysis, including geomapping, was performed in R Studio version 4.1.2 (R Core Team, 2021). The original shapefile of Danish provinces was retrieved from the European Commission – Eurostat (Eurostat, 2021).

In accordance with Danish law, no informed consent was required for this study to further qualify our national epidemiological monitoring using anonymized routine surveillance data. The planning and conduct of this study are in line with the Declaration of Helsinki, as revised in 2013.

## 3 Results

### 3.1 Diagnostic testing

From 2012 to 2021, 7,783 tests were conducted by SSI on samples from 4,946 patients, amounting to an average of 1.6 tests per patient. The average number of tests performed per year over the study period was 778, with a range of 593 tests in 2012 to 1172 tests in 2018.

### 3.2 Incidence of leptospirosis

**Figure 1:**
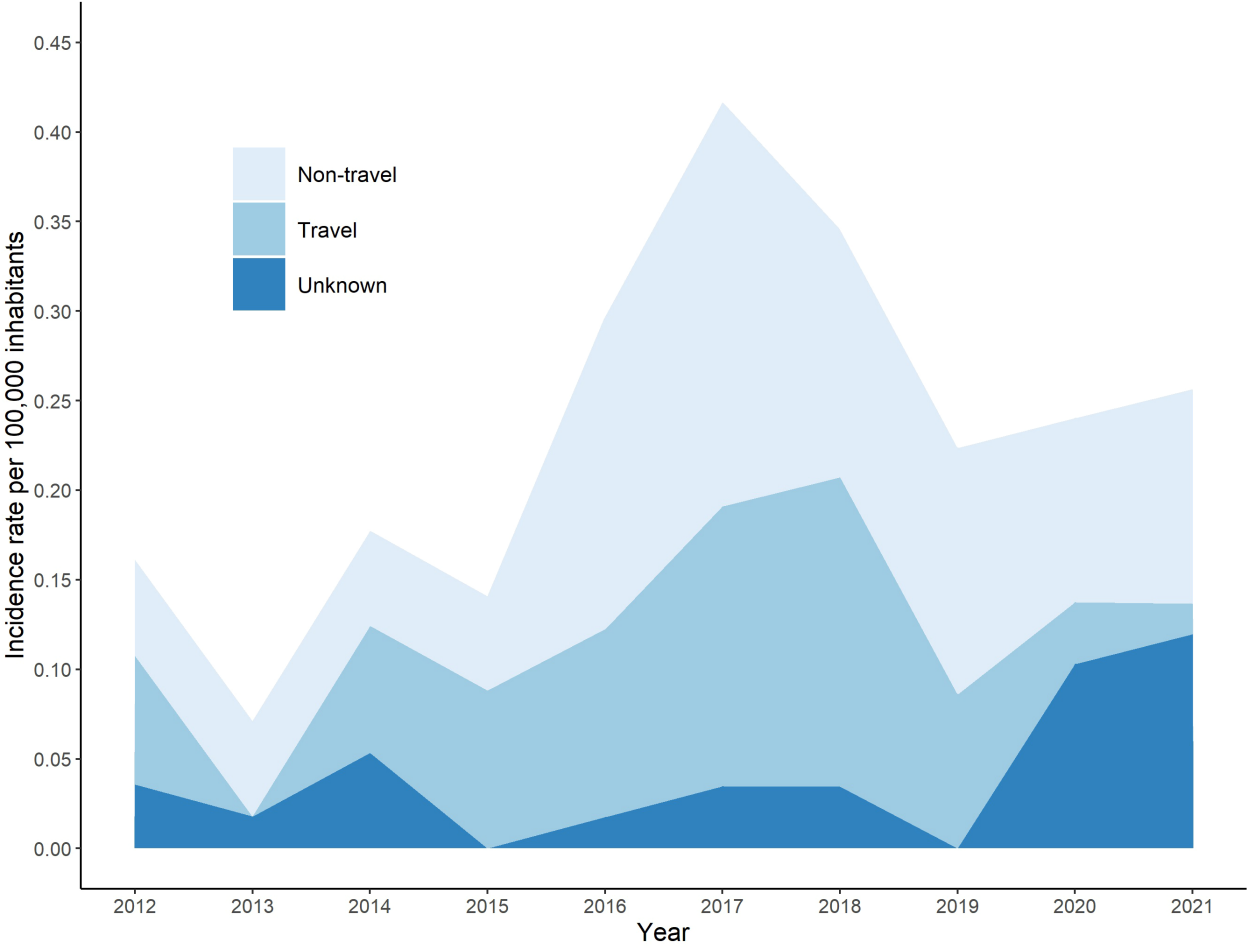
Annual incidence rate of leptospirosis per 100,000 inhabitants by year and reported travel-related exposure status in Denmark, 2012-2021.

From 2012 to 2021, 134 cases of leptospirosis were diagnosed in Denmark. **Figure 1** shows annual incidence rates per 100,000 inhabitants over the entire study period by reported travel status at the time of infection (data regarding the number of cases reported per year can be found in **Supplementary Table 1**). The year with the highest incidence rate was 2017 at 0.42 (24 cases), followed by 2018, which had an incidence rate of 0.35 (20 cases) and the highest number of travel-related cases reported throughout the study period. The lowest incidence rate observed was in 2013 at 0.07 (4 cases). Over the entire study period, the average number of annual cases was 13 and the overall incidence rate was 0.23 per 100,000 inhabitants.

**Figure 2:**
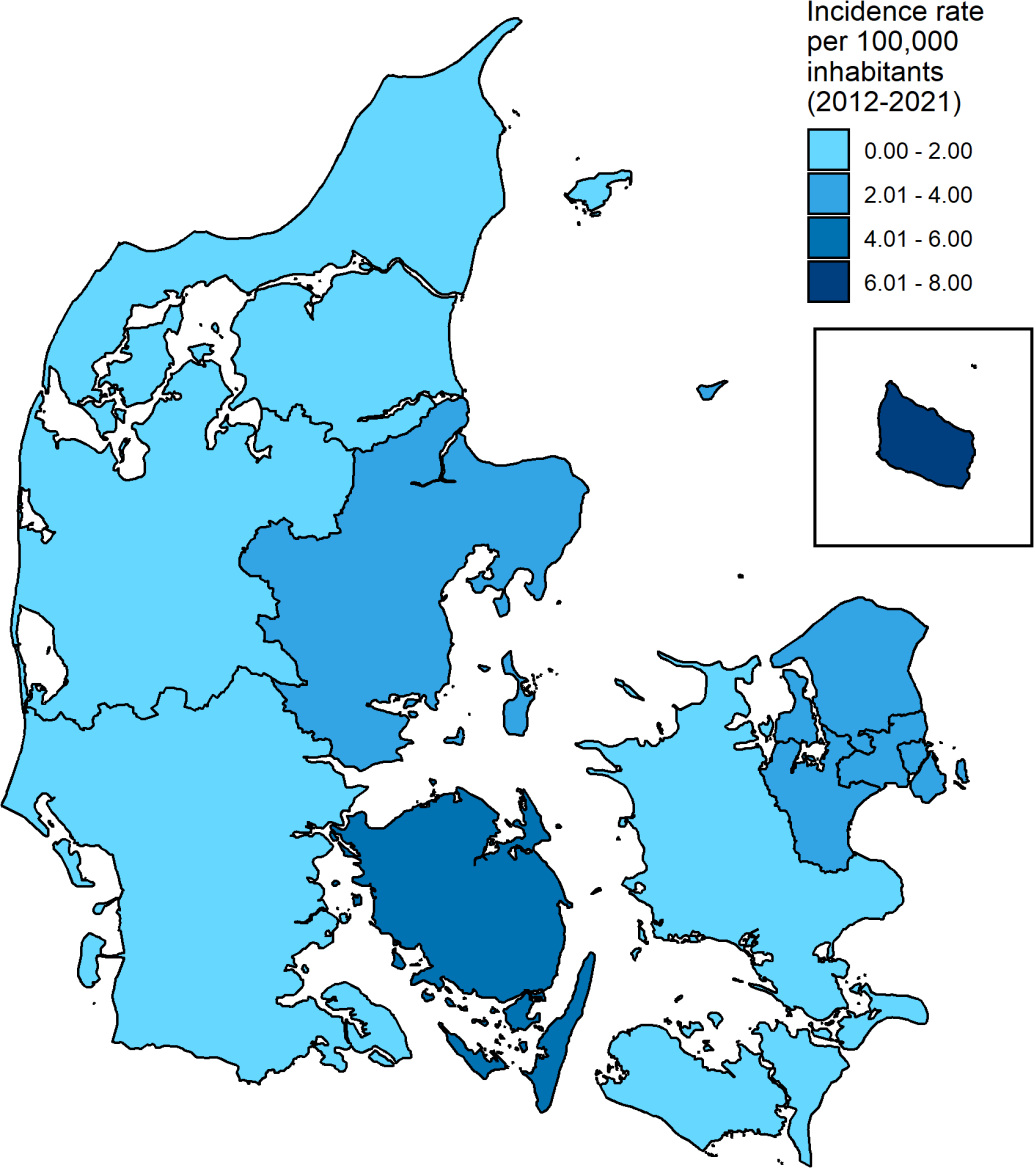
Geographical distribution by province of incidence rates per 100,000 of all cases over the entire study period in Denmark, 2012-2021.

**Figure 2** shows the geographic distribution of incidence rates of leptospirosis by province for all cases over the entire study period, regardless of travel status. The highest incidence rate was observed in Bornholm Province at 7.54 per 100,000 inhabitants. The lowest incidence rate of 0.85 per 100,000 inhabitants was observed in Nordjylland Province.

**Figure 3:**
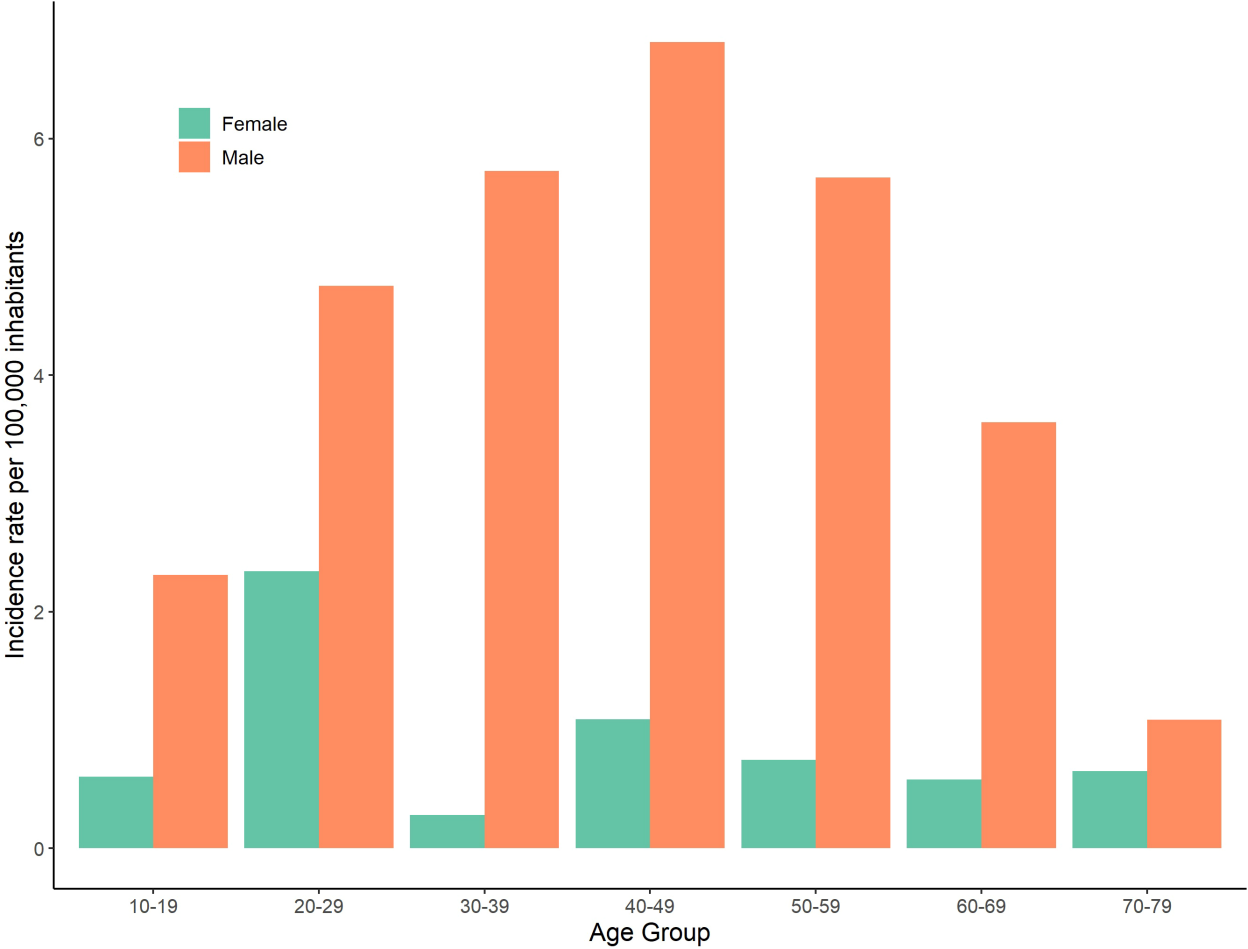
Incidence rates per 100,000 of leptospirosis in Denmark by age group and sex, 2012-2021.

Out of the 134 cases diagnosed over the study period, males accounted for 83% (111). The overall median age at the time of infection was 42, with a range from 10 to 77. **Figure 3** shows the average incidence rates by age group and sex over the entire study period. Men in age group 40-49 had the highest incidence rate (6.82 per 100,000) and the highest incidence rate among women was in the age group 20-29 (2.34 per 100,000).

### 3.3 Diagnostic tests, serology and PCR

**Table 1** shows the number of cases by serovar per year from 2012-2021. The most common serovar out of the 134 diagnosed cases of leptospirosis was Icterohaemorrhagiae with 34 (25%). Additionally, 54 cases (40%) had positive results for other serovars and 46 (34%) cases tested positive for leptospirosis *via* PCR only. Distribution of cases over the study period varied among the different serovars, though Icterohaemorrhagiae remained dominant across the study period.

**Table 1:** Reported cases leptospirosis by serovar and year, Denmark, 2012-2021.

Serovar	2012	2013	2014	2015	2016	2017	2018	2019	2020	2021	Total
Ballum					1						1
Bataviae									1		1
Canicola						2	1	1			4
Cynopteri							1				1
Grippotyphosa							2				2
Hebdomadis								2			2
Hurstbridge	1		1	1	2	2			6	5	18
Icterohaemorrhagiae	2	3	2	2	5	4	3	6	2	5	34
Patoc	2		3			2	4			2	13
*Leptospira* spp.*	4		3	4	6	11	7	4	5	2	46
Pomona					1		1				2
Sejroe		1	1	1	2	3	1			1	10
Total	9	4	10	8	17	24	20	13	14	15	134

*Serology undeterminable, individuals tested positive by PCR only.

**Figure 4:**
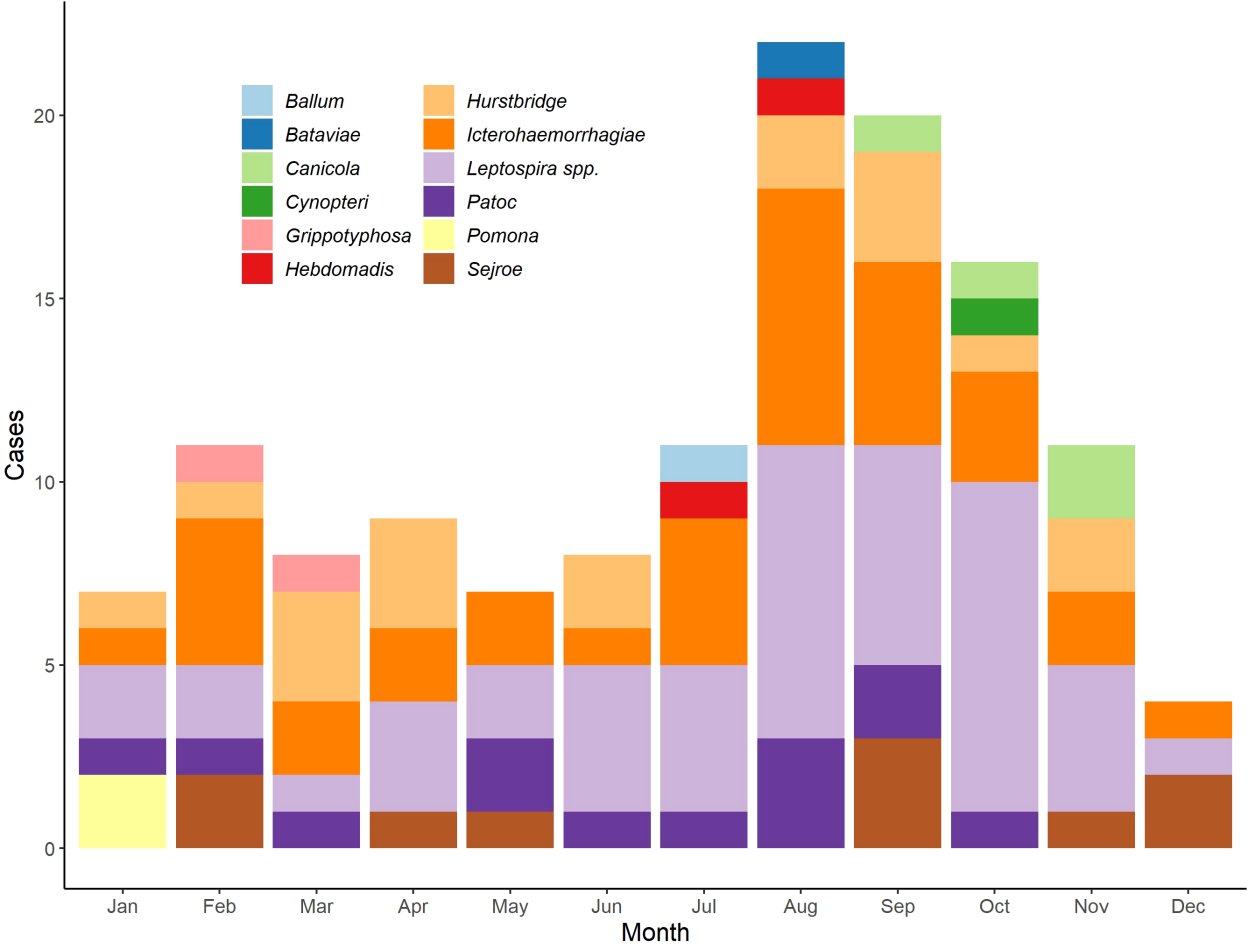
Distribution of serovars among cases of leptospirosis by month, Denmark, 2012-2021.

**Figure 4** shows the distribution of cases of leptospirosis by serovar (including those diagnosed *via* PCR only) and by month. The overall incidence was highest in August and September, as was the case for Icterohaemorrhagiae with seven and five cases, respectively.

### 3.4 Notifications

Of the 134 cases diagnosed between 2012 and 2021, 118 (88%) were clinically notified to the Department of Infectious Disease Epidemiology and Prevention at SSI. Of these 118 clinically notified cases, 58 (49%) were hospitalized with leptospirosis and three (2.5%) deaths were reported within the study period.

Among the 16 cases without a clinical notification, 12 (75%) were male and four (25%) were female. The age of the 16 non-notified cases resembled that of the notified cases, with the highest incidence in males between 30-39 (3, 25%) and females between 20-29 (2, 50%). Half of the 16 non-notified cases had unknown source of exposure due to missing information from the clinical notification, and half had extra information available in MiBA, with travel abroad being the most frequent source of exposure. The 16 non-notified cases closely resembled the other 118 cases of leptospirosis in Denmark, and were therefore included in all analyses.

### 3.5 Source of infection

**Table 2** describes reported sources of exposure by sex and select serovars. Travel abroad was the most common source of exposure with 46 (34%) cases total, with Asia being the most common geographical area of reported infection with 30 (65%) cases. Travel abroad also accounted for the most frequently reported source of infection among women with nine (39%) cases. Overall, work-related infection was the second most frequent route of exposure with 33 (71%) cases, where farmer/farming and sewage work were the most common exposure subgroups reported with 11 (33%) and 9 (27%) cases, respectively. Of the 33 work-related cases, 32 (97%) of them were men. Recreation had 21 (46%) cases, with contact with fresh water being the most common exposure subgroup with 10 (48%) cases and pet rats being the second most common subgroup with five (24%) cases. Eight cases reported other exposures, where direct contact with rats (non-domestic) accounted for seven (88%) cases. Lastly, 24 (18%) cases either had unknown exposure or no suspected source of exposure was reported.

**Table 2:** Main sources and subgroups of reported exposure of leptosporosis by sex and serovar in Denmark, 2012-2021 (N=134).

Exposures*	Total^ǂ^ (%)	Sex (%)		Serovar (%)					
		Female	Male	Hurst.^a^	IH^b^	Sejroe	Patoc^c^	Other^d^	PCR^e^
**Work-related**	**33 (25%)**	**1 (1%)**	**32 (24%)**	**3 (2%)**	**14 (10%)**	**2 (1%)**	**4 (3%)**	**3 (2%)**	**7 (5%)**
Contact with rats (non-farming)	2		2		1				1
Farmer/work with farm animals	11	1	10	2	2	2	1	1	3
Fish farming	4		4	1	2			1	
Other/unknown	7		7		4		2	1	
Sewage	9		9		5		1		3
**Recreation**	**21 (16%)**	**3 (2%)**	**18 (13%)**	**2 (1%)**	**8 (6%)**	**2 (1%)**			**9 (7%)**
Camping	1		1		1				
Contact with fresh water	10	1	9	1	4	1			4
Horses	1		1		1				
Pet rats	5	2	3		2	1			2
Swim-run	3		3						3
Water sports	1		1	1					
**Travel abroad**	**46 (34%)**	**9 (7%)**	**37 (28%)**	**5 (4%)**	**4 (3%)**	**2 (1%)**	**7 (5%)**	**5 (4%)**	**23 (17%)**
Africa	1		1				1		
Americas	8	1	7	1		1		2	4
Asia	30	7	23	3	3	1	4	3	16
Europe	3	1	2		1				2
Unknown	4		4	1			2		1
**Sewage-related (non-work)**	**2 (1%)**	**1 (1%)**	**1 (1%)**	**1 (1%)**					**1 (1%)**
Flooding	1	1		1					
Other	1		1						1
**Other**	**8 (6%)**	**4 (3%)**	**4 (3%)**		**2 (1%)**			**4 (3%)**	**2 (1%)**
Direct contact with rats	7	3	4		2			3	2
Visiting a farm	1	1						1	
Unknown^**^	**24 (18%)**	**5 (4%)**	**19 (14%)**	**7 (5%)**	**6 (4%)**	**4 (3%)**	**2 (1%)**	**1 (1%)**	**4 (3%)**
**Total**	**134 (100%)**	**23 (17%)**	**111 (83%)**	**18 (13%)**	**34 (25%)**	**10 (7%)**	**13 (10%)**	**13 (10%)**	**46 (34%)**

^*^All exposure groups with the exception of Travel abroad assume infection in Denmark.

^ǂ^Serum was considered positive when the titer was ≥100.

^**^Non-notified cases without further information.

a Hurstbridge.

b Icterohaemorrhagiae.

c Positive result for Patoc is considered a case due to the cross reactivity of Patoc with other serovars in addition to positive clinical assessment.

d Includes serovars Ballum, Bataviae, Canicola, Cynopteri, Grippotyphosa, and Hebdomadis.

e Includes cases diagnosed by Polymerase Chain Reaction (PCR) only. Bold values indicate the 6 main exposure categories (plus the total) and the corresponding subcategories are listed under each bolded category.

Icterohaemorrhagiae was the most common serovar among cases with work-related and recreational exposure with 14 (42%) and eight (38%) cases, respectively. Among those with work-related infection with Icterohaemorrhagiae, the most common source of exposure was among those working with sewage with five (33%) cases. Hurstbridge was the most common serogroup among cases with unknown source of exposure with seven (29%) cases. Patoc was the most common test result among travel-related cases with 7 (15%) cases.

## 4 Discussion

### 4.1 Main findings

The average incidence per year was 13 cases, a substantial drop from 17 cases between 1980 – 2012 (van Alphen et al., 2015), which can be attributed to several factors. Firstly, fish farming, which was the most frequent source of exposure among notified cases between 1980-2012 (37/170, 48.2%) (van Alphen et al., 2015), is an industry in Denmark that over the past 10 years has become highly regulated with many environmental restrictions. There were four reported infections among fish farmers during the current study period, further supporting that the decrease in traditional fish farming in Denmark has affected leptospirosis incidence since 2012. Additionally, the incidence rate among travel-related infections declined in 2020 and 2021, potentially due to restrictions on international travel that were in place at the time due to the COVID-19 pandemic, limiting travel-related exposures (Sykes et al., 2022).

The highest annual incidence rate of leptospirosis within the current study period was 0.42 per 100,000 inhabitants in 2017 (see **Figure 1**), where 24 cases were diagnosed. This could in part be attributed to a swim-run race that took part in Jutland in September, in which four athletes were subsequently infected with leptospirosis after swimming in a fresh water stream and running in muddy conditions over a field after harvest (Anonymous, 2018). Similar observations have been made in other parts of the world as well, with infection occurring among athletes and recreational water-sport participants taking part in fresh water activities (Sejvar et al., 2003; Wasiński and Dutkiewicz, 2013; Walker, 2018). The Danish swim-run also coincided with the month over the entire study period in which most cases were observed, which is also in line with seasonality of leptospirosis observed in the past (see **Figure 4**) (Holk et al., 2000; van Alphen et al., 2015). A high overall incidence rate was observed in Bornholm Province (see **Figure 2**), which can be attributed to the low population over the study period, despite three cases being observed in total.

Contrarily, the lowest incidence rate observed it the current study period was 0.07 per 100,000 inhabitants in 2013 with four cases. All four of these cases were men and half were in the 50-59 age group, one in 20-29 and one in 10-19. Two of the cases had work-related exposure, one was exposed *via* recreational activity and one had an unknown source of exposure. Additionally, the cases came from different provinces across Denmark and do not differ substantially from the rest of the study population.

This study showed a shift in reported source of exposure among those diagnosed with leptospirosis compared to past studies. In the current study period, the most common sources of reported exposure shifted to work-related exposures among farmers and sewage workers as well as those with recreational contact with fresh water, not including swim-runs and water sports (see **Table 2**). Additionally, direct contact with rats was a common source of exposure in the current study period, a trend previously highlighted by Brockmann et al. (2016). The combination of transmission from animals, primarily rats, and environmental aspects, like contamination of fresh water sources and severe weather events such as excessive rain and flooding, highlights the need for a One Health approach when conducting leptospirosis surveillance.

Despite some variations in certain aspects of disease epidemiology, sex and age group of cases remained relatively consistent between the previous study period from 1980-2012 and the current study period, with men between the ages of 40-59 being the most common groups reportedly infected (see **Figure 3**) (van Alphen et al., 2015). The median age of infection in the previous study was 49 (van Alphen et al., 2015) and in this study period was 42, highlighting similar demographics among cases.

### 4.2 Implications

In Denmark, if a patient tests positive for leptospirosis *via* PCR, submission of a follow up sample is requested in order to conduct serological testing. Serovar identification is paramount in the search for the most probable route of exposure. Specific serovars colonize and adapt to different small rodents and other mammals. For example, over the last several decades in Denmark, the most common serovar has been *L. interrogans* serovar Icterohaemorrhagiae (see **Table 1**), which is often found in the urine of rats (Department of Infectious Disease and Prevention, 2016). Additionally, in Denmark, Pomona is commonly found in mice and pigs, and Hardjo in cattle (Cilia et al., 2020; Haji Hajikolaei et al., 2022). However, obtaining additional samples is not always possible, leading to the diagnosis of some patients using PCR only . In order to best follow and understand serologic trends of leptospirosis in both the human and animal populations, it is important to emphasize the submission of a second sample for further testing. Given the zoonotic nature of the disease, it is essential to collaborate with wild life consultants, pest controllers and those working in agriculture when focusing on preventative measures.

### 4.3 Limitations and strengths

Disease surveillance, including that of leptospirosis, is highly digitalized in Denmark, lending itself to complete and reliable data that is often readily available. However, there are certain limitations to this study. One limitation is that undiagnosed and/or mild cases of leptospirosis are not captured by routine surveillance. Therefore, leptospirosis surveillance is more prone to highlight the more severe cases that are often hospitalized, potentially skewing the observed trends of disease. A second limitation is that not all laboratory confirmed cases of leptospirosis during the study period were notified to SSI, leading to a lack of reported exposure and travel history for certain patients. However, the overwhelming majority of cases (88%) were notified to SSI in a timely manner, a substantial increase from 30% during the period of 1980-2012 (van Alphen et al., 2015). Thus, we need to increase the awareness of leptospirosis among the medical community to ensure as complete reporting as possible.

### 4.4 Opportunities for future research

Further research on this topic could investigate the incidence and severity of Weil’s disease in Denmark and remaining sequelae in the months following recovery. Additionally, as water sports and other fresh water-based recreational activities increase in popularity in Denmark, further research could focus on this relatively new and unique source of exposure and potential prevention strategies. Future studies could also investigate whether *L. fainei* can be attributed to human leptospirosis infection by sequencing the 16S amplification. Finally, seroprevalence studies in e.g., rats and domestic animals could be useful in estimating the risk of human infection.

### 4.5 Conclusion

The decline in the annual incidence rate of leptospirosis over the past several decades, from previous studies to the current study period of 2012-2021, highlights that leptospirosis is a rare, sporadic disease in Denmark. Despite this fall, overall incidence has been steady over the current study period, further highlighting the importance of leptospirosis surveillance, as it has the ability to cause severe disease and has a high rate of hospitalization. Additionally, disease surveillance in support of up-to-date prevention measures is imperative, including guidance for those with significant occupational risk and travelers with possible exposure, e.g. journeys to Asia. Surveillance from a One Health perspective, utilizing data regarding trends in disease among animals as well as environmental data, is critical to get a complete picture of leptospirosis, predicting outbreaks before they happen, and strategizing their prevention.

## Data availability statement

The original contributions presented in the study are included in the article/**Supplementary Material**. Further inquiries can be directed to the corresponding author.

## Author contributions

CE was principal author. CE, CK and GB responsible for epidemiological data analyses. CJ and KK responsible for diagnostic laboratory analyses. All authors contributed to the writing and editing of manuscript. All authors critically reviewed and approved the manuscript for publication and agree to be accountable for the final version.

## References

[B1] The Danish Health Authority.(2000). Bekendtgørelse om lægers anmeldelse af smitsomme sygdomme m.v. (Copenhagen, Denmark).

[B2] Anonymous. (2018). Annual Report on Zoonoses in Denmark 2017. (Copenhagen, Denmark: National Food Institute, Technical University of Denmark).

[B3] Eurostat. (2021). Home, GISCO, Geodata, reference data, Administrative Units / Statistical units, NUTS, 1M, EPSG: 3035 shapefile. Administrative boundaries: © EuroGeographics, © TurkStat. Source: European Commission – Eurostat/GISCO. Online. Available at: https://ec.europa.eu/eurostat/web/gisco/geodata/reference-data/administrative-units-statistical-units/nuts#nuts21 (accessed on: 28/12/2022). Available at: https://ec.europa.eu/eurostat/web/gisco/geodata/reference-data/administrative-units-statistical-units/nuts.

[B4] Brockmann S. O. Ulrich L. Piechotowski I. Wagner-Wiening C. Nöckler K. Mayer-Scholl A.. (2016). Risk factors for human leptospira seropositivity in south Germany. SpringerPlus. 5 (1), 1796. doi: 10.1186/s40064-016-3483-8 27803844 PMC5069215

[B5] Centers for Disease Control and Prevention National Center for Emerging and Zoonotic Infectious Diseases (NCEZID) (2015). “Division of high-consequence pathogens and pathology (DHCPP),” in Risk of exposure. Available at: https://www.cdc.gov/leptospirosis/exposure/index.html.

[B6] Cilia G. Bertelloni F. Fratini F. (2020). Leptospira infections in domestic and wild animals. Pathogens. 9 (7), 573. doi: 10.3390/pathogens9070573 32679834 PMC7400056

[B7] Costa F. Hagan J. E. Calcagno J. Kane M. Torgerson P. Martinez-Silveira M. S.. (2015). Global morbidity and mortality of leptospirosis: A systematic review. PloS Negl. Trop. Dis. 9 (9), e0003898. doi: 10.1371/journal.pntd.0003898 26379143 PMC4574773

[B8] Danish Veterinary and Food Administration (2019). Leptospirose. (Copenhagen, Denmark: The Ministry of Food, Agriculture and Fisheries of Denmark). Available from: https://www.foedevarestyrelsen.dk/Leksikon/Sider/Leptospirose.aspx.

[B9] Haake D. A. Levett P. N. (2015). Leptospirosis in humans. Curr. Top. Microbiol. Immunol. 387, 65–97. doi: 10.1007/978-3-662-45059-8_5 25388133 PMC4442676

[B10] Haji Hajikolaei M. R. Rezaei S. Ghadrdan Mashhadi A. R. Ghorbanpoor M. (2022). The role of small ruminants in the epidemiology of leptospirosis. Sci. Rep. 12 (1), 2148. doi: 10.1038/s41598-022-05767-x 35140240 PMC8828929

[B11] Holk K. Nielsen S. V. Rønne T. (2000). Human leptospirosis in Denmark 1970-1996: an epidemiological and clinical study. Scand. J. Infect. Dis. 32 (5), 533–538. 10.1080/003655400458839 11055660

[B12] Levett P. N. (2003). Usefulness of serologic analysis as a predictor of the infecting serovar in patients with severe leptospirosis. Clin. Infect. Diseases. 36 (4), 447–452. doi: 10.1086/346208 12567302

[B13] Pan American Health Organization Leptospirosis. Available at: https://www.paho.org/en/topics/leptospirosis.

[B14] Picardeau M. (2013). Diagnosis and epidemiology of leptospirosis. Med. Mal Infect. 43 (1), 1–9. doi: 10.1016/j.medmal.2012.11.005 23337900

[B15] Rajapakse S. (2022). Leptospirosis: clinical aspects. Clin. Med. (Lond). 22 (1), 14–17. doi: 10.7861/clinmed.2021-0784 PMC8813018 35078790

[B16] R Core Team (2021). R: A language and environment for statistical computing (Vienna, Austria: R Foundation for Statistical Computing).

[B17] Sejvar J. Bancroft E. Winthrop K. Bettinger J. Bajani M. Bragg S.. (2003). Leptospirosis in “Eco-challenge” athletes, Malaysian Borneo, 2000. Emerg. Infect. Dis. 9 (6), 702–707. doi: 10.3201/eid0906.020751 12781010 PMC3000150

[B18] Department of Infectious Disease and Prevention. (2016) EPI-NEWS No 28-33 - 2016. (Copenhagen, Denmark: Statens Serum Institut) Available from: https://en.ssi.dk/news/epi-news/2016/no-28-33---2016.

[B19] Department of Infectious Disease and Prevention. (2020). Leptospirosis 2019. (Copenhagen, Denmark: Statens Serum Institut). Available from: https://en.ssi.dk/surveillance-and-preparedness/surveillance-in-denmark/annual-reports-on-disease-incidence/leptospirosis-2019.

[B20] Statens Serum Institut (2022) Mandatory notification systems. (Copenhagen, Denmark: Statens Serum Institut). Available from: https://en.ssi.dk/surveillance-and-preparedness/surveillance-in-denmark/mandatory-notification-systems.

[B21] Statistics Denmark (2012-2021). Population Figures. (Copenhagen, Denmark). Available from: https://www.dst.dk/da/Statistik/emner/borgere/befolkning/befolkningstal

[B22] Sykes J. E. Haake D. A. Gamage C. D. Mills W. Z. Nally J. E. (2022). A global one health perspective on leptospirosis in humans and animals. J. Am. Vet. Med. Assoc. 260 (13), 1589–1596. doi: 10.2460/javma.22.06.0258 35895801

[B23] van Alphen L. B. Lemcke Kunoe A. Ceper T. Kähler J. Kjelsø C. Ethelberg S.. (2015). Trends in human leptospirosis in Denmark, 1980 to 2012. Eurosurveillance. 20 (4), 21019. doi: 10.2807/1560-7917.ES2015.20.4.21019 25655055

[B24] Villumsen S. Pedersen R. Borre M. B. Ahrens P. Jensen J. S. Krogfelt K. A. (2012). Novel TaqMan^®^ PCR for detection of leptospira species in urine and blood: pit-falls of in silico validation. J. Microbiol. Methods 91 (1), 184–190. doi: 10.1016/j.mimet.2012.06.009 22750039

[B25] Villumsen S. Pedersen R. Krogfelt K. A. Jensen J. S. (2010). Expanding the diagnostic use of PCR in leptospirosis: Improved method for DNA extraction from blood cultures. PloS One 5 (8), e12095. doi: 10.1371/journal.pone.0012095 20711446 PMC2920309

[B26] Vincent A. T. Schiettekatte O. Goarant C. Neela V. K. Bernet E. Thibeaux R.. (2019). Revisiting the taxonomy and evolution of pathogenicity of the genus leptospira through the prism of genomics. PloS Negl. Trop. Dis. 13 (5), e0007270. doi: 10.1371/journal.pntd.0007270 31120895 PMC6532842

[B27] Voldstedlund M. Haarh M. Mølbak K. (2014). The Danish Microbiology Database (MiBa) 2010 to 2013. Eurosurveillance 19 (1), 20667. doi: 10.2807/1560-7917.ES2014.19.1.20667 24434175

[B28] Walker M. D. (2018). Leptospirosis: the possible risk to those participating in water-based sports and activities. Br. J. Gen. Pract. 68 (673), 394–395. doi: 10.3399/bjgp18X698285 30049778 PMC6058646

[B29] Wasiński B. Dutkiewicz J. (2013). Leptospirosis – current risk factors connected with human activity and the environment. Ann. Agric. Environ. Med. 20 (2), 239–244. 23772568

[B30] World Health Organization (2003). Human Leptospirosis: Guideline for Diagnosis, Surveillance and Control.(Malta: World Health Organization), 122p.

