# Supplementary material for: Trends in human leptospirosis in Denmark, 2012-2021
Source: Front Cell Infect Microbiol. 2023 Feb 13;13:1079946. doi: 10.3389/fcimb.2023.1079946 (PMC9968856; doi:10.3389/fcimb.2023.1079946)
Supplement: Supplementary file 1 [file Table_1.docx]

Supplementary material

Table 1. Reported cases and incidence rates per year overall and by main and subgroups of exposure, Denmark, 2012-2021 (N=134)”

| **Exposure** | **Year (IR)** | | | | | | | | | | **Total** |
| --- | --- | --- | --- | --- | --- | --- | --- | --- | --- | --- | --- |
|  | **2012** | **2013** | **2014** | **2015** | **2016** | **2017** | **2018** | **2019** | **2020** | **2021** |  |
| **Total cases** | **9 (0.16)** | **4 (0.07)** | **10 (0.18)** | **8 (0.14)** | **17 (0.30)** | **24 (0.42)** | **20 (0.35)** | **13 (0.22)** | **14 (0.24)** | **15 (0.26)** | **134** |
| **Work-related** | **2 (0.04)** | **2 (0.04)** | **1 (0.02)** | **1**  **(0.02)** | **4 (0.07)** | **5 (0.09)** | **3 (0.05)** | **5 (0.09)** | **5 (0.09)** | **5 (0.09)** | **33** |
| Contact with rats - non-farming |  |  |  |  |  |  |  | 1 | 1 |  | **2** |
| Farmer/farm animals | 1 |  |  | 1 | 1 | 1 | 1 | 2 | 2 | 2 | **11** |
| Fish farming |  | 1 |  |  | 1 |  | 1 |  |  | 1 | **4** |
| Other/unknown |  |  |  |  | 1 | 3 |  | 1 |  | 2 | **7** |
| Sewage | 1 | 1 | 1 |  | 1 | 1 | 1 | 1 | 2 |  | **9** |
| **Recreation** | **1 (0.02)** | **1 (0.02)** | **1 (0.02)** | **2 (0.04)** | **5 (0.09)** | **7 (0.12)** | **1 (0.02)** | **2 (0.03)** |  | **1 (0.02)** | **21** |
| Camping |  |  |  |  | 1 |  |  |  |  |  | **1** |
| Contact with water | 1 | 1 | 1 |  | 2 | 3 |  | 1 |  | 1 | **10** |
| Horses |  |  |  |  |  |  |  | 1 |  |  | **1** |
| Pet rats |  |  |  | 1 | 2 | 1 | 1 |  |  |  | **5** |
| Swim-run |  |  |  | 1 |  | 2 |  |  |  |  | **3** |
| Water sports |  |  |  |  |  | 1 |  |  |  |  | **1** |
| **Travel abroad** | **4 (0.07)** |  | **4 (0.07)** | **5 (0.09)** | **6 (0.10)** | **9 (0.16)** | **10 (0.17)** | **5 (0.09)** | **2**  **(0.03)** | **1 (0.02)** | **46** |
| Africa |  |  |  |  |  | 1 |  |  |  |  | **1** |
| Americas |  |  | 1 | 2 | 2 | 1 |  | 1 | 1 |  | **8** |
| Asia | 1 |  | 3 | 2 | 4 | 7 | 9 | 3 | 1 |  | **30** |
| Europe | 1 |  |  | 1 |  |  | 1 |  |  |  | **3** |
| Unknown | 2 |  |  |  |  |  |  | 1 |  | 1 | **4** |
| **Sewage-related** |  |  | **1 (0.02)** |  |  | **1 (0.02)** |  |  |  |  | **2** |
| Flooding |  |  | 1 |  |  |  |  |  |  |  | **1** |
| Other |  |  |  |  |  | 1 |  |  |  |  | **1** |
| **Other** |  |  |  |  | **1 (0.02)** |  | **4 (0.07)** | **1 (0.02)** | **1 (0.02)** | **1 (0.02)** | **8** |
| Direct contact with rats |  |  |  |  | 1 |  | 4 | 1 |  | 1 | **7** |
| Farm (visiting) |  |  |  |  |  |  |  |  | 1 |  | **1** |
| **Unknown** | **2**  **(0.04)** | **1 (0.02)** | **3**  **(0.05)** |  | **1 (0.02)** | **2**  **(0.03)** | **2**  **(0.03)** |  | **6 (0.10)** | **7 (0.12)** | **24** |
